# Donepezil Ameliorates Pulmonary Arterial Hypertension by Inhibiting M2-Macrophage Activation

**DOI:** 10.3389/fcvm.2021.639541

**Published:** 2021-03-15

**Authors:** Haihua Qiu, Yibo Zhang, Zhongyu Li, Ping Jiang, Shuhong Guo, Yi He, Yuan Guo

**Affiliations:** ^1^Department of Cardiovascular Medicine, The Affiliated Zhuzhou Hospital Xiangya Medical College, Central South University, Zhuzhou, China; ^2^Department of Ultrasound, The Affiliated Zhuzhou Hospital Xiangya Medical College, Central South University, Zhuzhou, China; ^3^Laboratory Medicine Center, The Affiliated Zhuzhou Hospital Xiangya Medical College, Central South University, Zhuzhou, China; ^4^Department of Cardiovascular Medicine, Xiangya Hospital, Central South University, Changsha, China

**Keywords:** donepezil, pulmonary arterial hypertension, cholinesterase inhibitor, M2-macrophage, pulmonary arterial smooth muscle cells

## Abstract

**Background:** The beneficial effects of parasympathetic stimulation in pulmonary arterial hypertension (PAH) have been reported. However, the specific mechanism has not been completely clarified. Donepezil, an oral cholinesterase inhibitor, enhances parasympathetic activity by inhibiting acetylcholinesterase, whose therapeutic effects in PAH and its mechanism deserve to be investigated.

**Methods:** The PAH model was established by a single intraperitoneal injection of monocrotaline (MCT, 50 mg/kg) in adult male Sprague-Dawley rats. Donepezil was administered via intraperitoneal injection daily after 1 week of MCT administration. At the end of the study, PAH status was confirmed by echocardiography and hemodynamic measurement. Testing for acetylcholinesterase activity and cholinergic receptor expression was used to evaluate parasympathetic activity. Indicators of pulmonary arterial remodeling and right ventricular (RV) dysfunction were assayed. The proliferative and apoptotic ability of pulmonary arterial smooth muscle cells (PASMCs), inflammatory reaction, macrophage infiltration in the lung, and activation of bone marrow-derived macrophages (BMDMs) were also tested. PASMCs from the MCT-treated rats were co-cultured with the supernatant of BMDMs treated with donepezil, and then, the proliferation and apoptosis of PASMCs were evaluated.

**Results:** Donepezil treatment effectively enhanced parasympathetic activity. Furthermore, it markedly reduced mean pulmonary arterial pressure and RV systolic pressure in the MCT-treated rats, as well as reversed pulmonary arterial remodeling and RV dysfunction. Donepezil also reduced the proliferation and promoted the apoptosis of PASMCs in the MCT-treated rats. In addition, it suppressed the inflammatory response and macrophage activation in both lung tissue and BMDMs in the model rats. More importantly, donepezil reduced the proliferation and promoted the apoptosis of PASMCs by suppressing M2-macrophage activation.

**Conclusion:** Donepezil could prevent pulmonary vascular and RV remodeling, thereby reversing PAH progression. Moreover, enhancement of the parasympathetic activity could reduce the proliferation and promote the apoptosis of PASMCs in PAH by suppressing M2-macrophage activation.

## Introduction

Pulmonary arterial hypertension (PAH) is a life-threatening disease characterized by progressive pulmonary vascular remodeling and increased right ventricular (RV) afterload, which eventually leads to right heart failure (1). In PAH, the pulmonary vasculature is dynamically obstructed by vasoconstriction and structurally obstructed by adverse vascular remodeling (2). Various factors could aggravate the course of PAH, including vasoconstriction, thrombosis, and inflammation (3). Furthermore, excessive proliferation and apoptotic resistance of PASMCs could directly lead to the narrowing of the pulmonary vasculature, which also has been recognized as the typical reason for PAH deterioration (2, 4). Thus, exploring the factors and mechanisms leading to the abnormal proliferation and apoptosis of PASMCs may be beneficial for PAH therapy.

Evidence from many clinical and basic studies suggests that inflammatory injury plays a causal role in the pathogenesis of pulmonary vascular remodeling and PAH (2, 5). The infiltration of inflammatory and immune cells, such as macrophages, lymphocytes, dendritic cells, and mast cells, has been identified in the pulmonary tissue of patients with PAH (6). In particular, macrophages and macrophage-derived inflammatory mediators are essential for PAH progression (7). Recently, Pugliese et al. (8) observed the accumulation of cluster of differentiation 68^+^ (CD68^+^) and F4/80^+^ macrophages in the perivascular lung tissue in a mouse model of early-stage hypoxia-induced PAH through qualitative histologic and flow cytometry approaches, respectively. This indicated that robust recruitment and activation of macrophages as well as the production of an array of inflammatory factors play an important role in aggravating pulmonary artery remodeling and PAH (8, 9). Thus, inhibiting perivascular macrophage recruitment and the pulmonary inflammatory response could be an approach for reversing the course of PAH.

In general, macrophages can be categorized as classically activated (M1) and alternatively activated (M2) macrophages according to different environmental signals (10). M1-macrophages are known to amplify inflammation by promoting the secretion of proinflammatory factors, while M2-macrophages are thought to promote proliferation and angiogenesis (10). Interestingly, recent studies have observed that both M1- and M2-macrophages were activated in PAH patients and animal models of PAH; there was a predominance of M2-macrophage activation (11–13). Furthermore, M2-macrophages have been found to play a primary role in PAH progression by inducing inflammation and pulmonary vascular remodeling (11–13). Therefore, mediators for inhibiting macrophage activity may be a promising measure for PAH treatment.

Sympathetic and parasympathetic abnormalities have been well-identified in PAH, which is characterized by sympathetic overactivity and parasympathetic withdrawal (14, 15). Enhancing parasympathetic and reducing sympathetic activities can help reverse PAH progression (14, 15). Yoshida et al. (15) elucidated the therapeutic effects of electrical vagal nerve stimulation in a rat model of SU5416/hypoxia-induced PAH and speculated that they were related to the reduction of proinflammatory cytokine invasion. Further da Silva Goncalves Bos et al. (16) found that enhancement of parasympathetic activity by the acetylcholinesterase (AchE) inhibitor pyridostigmine improved survival, RV function, and pulmonary vascular remodeling in a rat model of SU-5416/hypoxia-induced PAH via its antiproliferative and anti-inflammatory effects. However, how parasympathetic activation induces antiproliferative and anti-inflammatory effects on PAH has not been elucidated and needs further exploration.

Generally, parasympathetic activation exerts protective effects in cardiopulmonary diseases, mainly via the “cholinergic anti-inflammatory pathway,” which also refers to the parasympathetic nervous system, the main cholinergic nerve system that controls innate immune responses to a variety of biological and chemical factors (17). Recent studies showed that enhancement of parasympathetic activity with stimulation of the anti-inflammatory cholinergic pathway could contribute to the improvement of various cardiovascular diseases, which also related to M2-macrophage activation (18, 19). Abid et al. (20) used murine M2-macrophages and co-cultured them with PASMCs obtained from patients with idiopathic PAH *in vitro* and discovered the ability of M2-macrophages to regulate PASMC function, indicating that M2-macrophage activation exacerbates pulmonary arterial remodeling and PAH progression. However, whether parasympathetic activation could inhibit M2-macrophages and thereby repress PASMC proliferation and PAH progression is unknown.

Therefore, this study is designed to evaluate whether parasympathetic activation by donepezil (DON), an AchE inhibitor, could improve monocrotaline (MCT)-induced PAH in rats. In this study, we assessed DON could ameliorate PASMC abnormalities by enhancing parasympathetic activity. Furthermore, we also assessed the inflammatory response, M2-macrophage activation, and the underlying mechanisms of M2-macrophages regulating PASMCs after DON treatment. Thus, this study aimed to provide more evidence and a potential therapeutic strategy for PAH.

## Materials and Methods

### Animals

Male Sprague Dawley (SD) rats (180–220 g) were randomly divided into three groups: control group, MCT group and DON group (MCT plus donepezil, *n* = 15 in each group). The PAH model was induced by a single intraperitoneal injection (50 mg/kg) of MCT (Sigma-Aldrich, USA) as our previous study described (21). Rats in control group were given equal volume of saline by intraperitoneal injection. MCT-induced PAH rats were intraperitoneal injected with DON (1 mg/kg, Selleck, USA) daily after 1-week delivery of MCT to the end of the fourth week (22, 23). All animal care and experiments were approved by the Animal Research Committee of Central South University in Hunan, China.

### Measurement of Echocardiography

Echocardiography was used to evaluate the structure and function of RV at the end of the experiment. Echocardiographic evaluation was measured by transthoracic echocardiography using a 15 MHz phased array transducer (Philips IE Elite). The indicators of diastolic and systolic thickness of the RV, and tricuspid annular plane systolic excursion (TAPSE) were measured as previously described (2).

### Assessment of Hemodynamics

The rats were anesthetized with 2.5% pentobarbital, the PE-50 catheter (inner diameter 0.58 mm, external diameter 0.96 mm) filled with heparinized saline was inserted into the RV and pulmonary artery via the jugular vein to measure RV systolic pressure (RVSP) and mean pulmonary artery pressure (mPAP). As previous study described, bend the front 1 cm of the PE-50 catheter into a small bend in 60°C water and immediately take into ice water to shape, the other end of which is connected to the regular pressure transducer through a syringe needle and calibrated. Then, incise the skin of right cervical area. Find the right external jugular vein and isolate it from the surrounding connective tissue. Use the silk suture to ligate the vein distally and then tie a loose knot proximally. Finally, after cutting a small “nick” proximal to the distal tied knot, inserting the catheter into the nick of the vein and gently pushing the catheter into the RV and then reached to the pulmonary arterial entrance via continuously adjusting the angle and depth of advancement of the catheter (24). The pressure was recorded by RM6240E instrument (Chengdu instrument factory, China).

### Histology of Heart and Lungs

After hemodynamic evaluation, heart and lung tissue of the rats were harvested, fixed by paraformaldehyde and paraffin-embedded, respectively. Hematoxylin and eosin (HE) staining were used to evaluate pulmonary arterial and RV remodeling. Percentage media thickness was measured to reflect pulmonary remodeling, which was calculated as: pulmonary wall thickness (WT) (%) = (external diameter–internal diameter)/external diameter × 100% (25). To calculate the RV hypertrophy index (RVHI) which reflects RV remodeling, the RV free wall was dissected from the left ventricle (LV) and ventricular septum (S). The RVHI was calculated by the weight ratio of the RV to the LV plus S [RVHI = RV/ (LV + S)] (26).

### Cholinergic System in Experimental PAH

AchE activity in plasma and lung tissue were measured by colorimetry according to the manufacturer's protocol (Acetylcholinesterase assay kit, A024-1-1, Nanjingjiancheng Bioengineering Institute, China).

### Immunohistochemistry Staining

Lung CD68 were stained by standard immunohistochemistry protocols to quantify the degree of macrophage infiltration. Briefly, CD68 expression levels were detected in paraffin embedded lung sections using anti-CD68 rat antibody (1:1000 dilution, Abcam, USA) (27). Cells positively stained for CD68 were automatically counted with image J software (NIH, Bethesda, MD, USA), which was calculated as the mean of 5 random fields from scanned images (DSAssistantLite software, Motic, China; ×400 magnification, image size: 715 × 408 μm). And the CD68 positive rate are calculated by CD68 positive cells to total cells in each field.

The proliferative and apoptotic abilities of PASMCs were, respectively, determined by immunohistochemical staining of lung section with Ki67 antibody (cat. no. GB111141, Servicebio, China) and TUNEL apoptosis assay kit (cat. no. G1507, Servicebio, China). Immunohistochemical staining was performed using the two-step immunohistochemical technique with diaminobenzidine (DAB, Sigma-Aldrich, USA) following the manufacturer's instructions. The positive stained cells were counted by image J software (NIH, Bethesda, MD, USA), and presented as the mean of 5 randomly selected pulmonary arteriole under ×400 magnification.

### Bone Marrow-Derived Macrophages (BMDMs) Isolation and Cultivation

BMDMs in the three groups were isolated and cultured using standard protocols (28). Intraperitoneally anesthetized rats with pentobarbital were fully sterilized with 75% ethanol. The femur and tibia were separated and isolated, and then the both ends of bones were cut and flushed with about 5 mL PBS repeatedly to bring the cells into single-cell suspension. Five Milliliter ACK red blood cell lysis buffer (Genview, China) was used to lyse the red blood cells for 2 min, and centrifuged at 1,500 rpm for 5 min. The supernatant was discarded and washed the cells twice with PBS. Finally, the BMDMs were obtained and cultured for 6–7 days in Dulbecco's Modified Eagle Medium (DMEM) medium containing 20% fetal bovine serum (FBS, Gbico, Invitrogen, USA), 1% penicillin-streptomycin and M-CSF (20 ng/mL, Peprotech, USA). Cell culture medium was replaced at the second and fourth days, respectively, and the adherent cells were considered as BMDMs. After reaching about 80% confluence, the cells were used in following experiments.

### Primary PASMCs Isolation and Cultivation

PASMCs were isolated from the pulmonary trunk of the rats as previously described (21). Briefly, rats were anesthetized with pentobarbital intraperitoneal injection and its pulmonary artery was separated from cardiopulmonary tissue under aseptic conditions. The outer membrane and endothelium of the pulmonary artery were carefully scraped off. The remaining smooth muscle was cut into 1 mm^3^ tissue fragments and placed in a 25 mL culture flask, which was cultivated with DMEM (Gibco, USA) containing 20% FBS (Gibco, USA) and 1% penicillin-streptomycin at an incubator (5% CO_2_, 37°C). Cells grew from the tissue sample after about 3 to 5 days. The identification of PASMCs was accomplished by immunofluorescence staining of α-smooth muscle actin (α-SMA) at passage 2 (Supplementary Figure 1). The cells of passage 3 to 6 at 80% confluence were used for further experiments.

### Proliferation and Apoptosis of PASMCs Assessment

#### 5-Ethynyl-20-Deoxyuridine (EdU) Assay

To evaluate the proliferation of PASMCs, EdU assay was performed using the BeyoClick™ EdU Cell Proliferation kit with Alexa Fluor 594 (cat. no. C0078S; Beyotime Institute of Biotechnology), according to the manufacturer's protocol (29). Positive cells were observed under an Olympus IX71 fluorescent microscope (Olympus Corp, Tokyo, Japan; magnification ×200) and analyzed using Image J software (NIH, Bethesda, MD, USA). EdU positive rate was calculated as the ratio of the mean numbers of EdU-positive cells to DAPI stained cells from 5 random fields in each group.

#### Terminal Deoxynucleotidyl Transferase dUTP Nick End Labeling (TUNEL) Assay

One Step TUNEL Apoptosis Assay Kit (cat. no. C1086; Beyotime Institute of Biotechnology) was used to evaluate the apoptosis rate of PASMCs according to the manufacturer's protocol (30). The fluorescence was observed under a fluorescence microscope (Olympus Corp, Tokyo, Japan) and the apoptosis rate of PASMCs was calculated as the ratio of TUNEL-positive cells to total cells in 5 randomly selected high-power field (magnification ×200) in each group.

### Co-incubation of PASMCs With the Supernatants of Primary BMDMs

Co-incubation was performed as previously described (31). As above mentioned, when the BMDMs reach about 80% confluence after cultivation for 6 days, 2 mL medium was used to cultivate for another 24 h. And then supernatant of BMDMs in the three groups were collected and separately incubated with the primary PASMCs of MCT group, which were also integration into about 80%. After 24 h co-incubation, proliferation, and apoptosis of PASMCs were assayed to evaluate the effects of BMDM-derived stimuli on its function.

### Western Blotting

As described in our previous work, western blotting was performed with rabbit polyclonal Nicotinic Acetylcholine Receptor alpha 7 (α-7nAchR) antibody (1:500, cat. no. ab10096, Abcam, USA), rabbit monoclonal NF-κB antibody (1:1000, cat. no.8242T, CST, USA), rabbit monoclonal β-actin (1:1000, cat. no. 4970S, CST, USA), and goat anti-rabbit (HRP) IgG antibody (1:5000, cat. no. ZB-2301, ZSGB-Bio, China) (32). ImageJ (NIH, Bethesda, MD, USA) was used to quantify the pixel intensities of immunoreactive bands.

### Real-Time Quantitative PCR

RNA extraction and real-time quantitative PCR were performed as our previously described (32). The sequences of the primer pairs used in this study were listed as follows. Data were normalized to β-actin and expressed as a relative ratio. Primer sequences for RT-PCR as follows.

**Table:** 

**Genes**		**Sequences**
IL-6	Sense	TCCTACCCCAATTTCCAATGCT
	Antisense	AACGCACTAGGTTTGCCGAG
IL-10	Sense	GGTTGCCAAGCCTTATCGGA
	Antisense	TCAGCTTCTCACCCAGGGAA
TNF-α	Sense	GATCGGTCCCCAAAGGGATG
	Antisense	TTTGCTACGACGTGGGCTAC
iNOs	Sense	GCTGCCAGGGTCACAACTTTA
	Antisense	CAGCTCAGTCCCTTCACCAA
MMP9	Sense	CCAGCCGACTTTTGTGGTCT
	Antisense	CTTCTCTCCCATCATCTGGGC
MRC-1	Sense	GTGGAGTGATGGAACCCCAG
	Antisense	CTGTCCGCCCAGTATCCATC
Arg-1	Sense	ACATTGGCTTGCGAGACGTA
	Antisense	ATCACCTTGCCAATCCCCAG
Fizz1	Sense	CTGCTACTGGGTGTGCTTGT
	Antisense	GCAGTGGTCCAGTCAACGAG
β-actin	Sense	ACTCTGTGTGGATTGGTGGC
	Antisense	CGCAGCTCAGTAACAGTCCG

### Statistical Analysis

Statistical analyses were performed using Prism for Windows (GraphPad 8 Software). Data were presented as mean ± SEM. All measured date were subjected to One-way ANOVA. Tukey's multiple comparisons was used to determine statistical significance of the simple effect between groups. *P* < 0.05 was considered statistically significant.

## Results

### DON Ameliorated Pulmonary Arterial Remodeling by Enhancing Parasympathetic Activity

**Figure 1:**
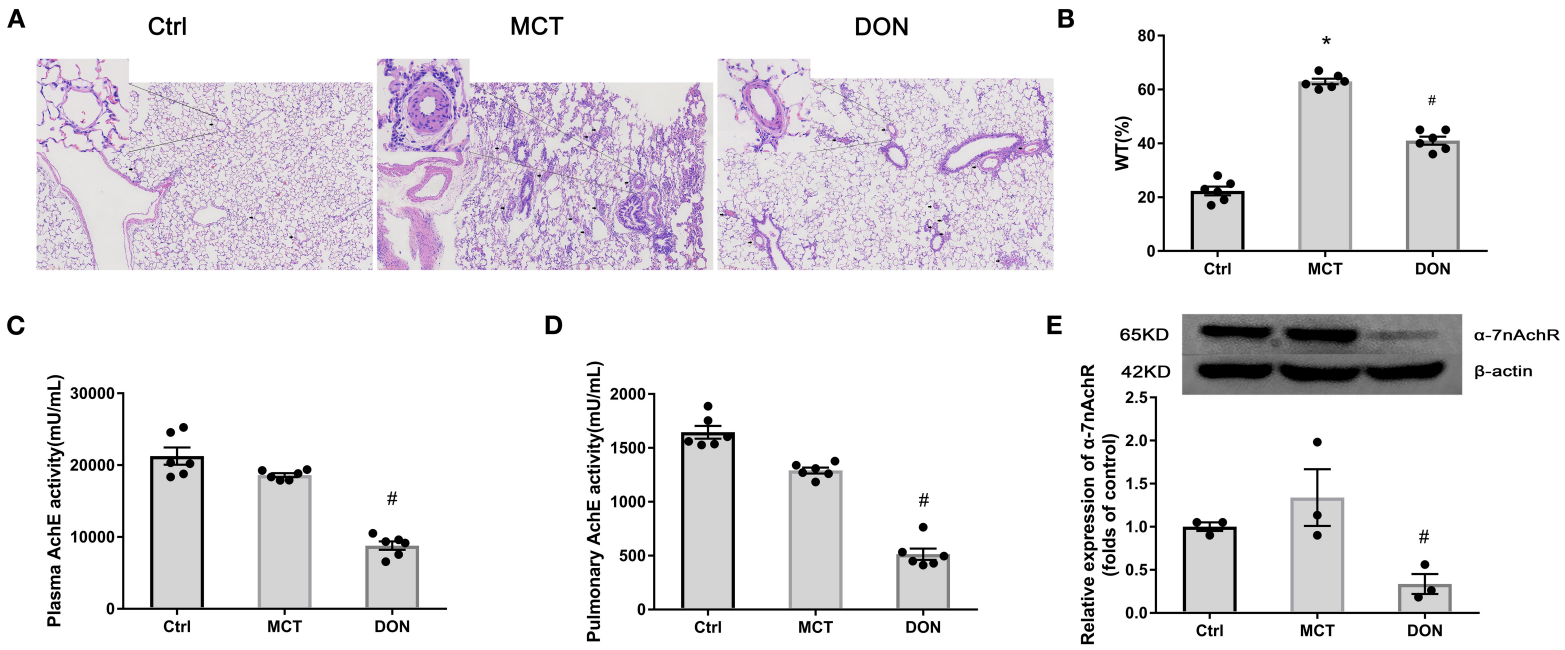
DON enhances parasympathetic activity and ameliorates pulmonary arterial remodeling. Lung tissue of MCT-induced rats after DON treatment were fixed and stained with HE at the end of the study. The pathological sections of lung tissue were scanned and analyzed by DSAssistantLite software (Motic, China; ×100 magnification, image size: 2,860 × 1,631 μm). Pulmonary arteriole from 5 random fields were analyzed. AchE from plasma and lung tissue were tested by colorimetry. Relative protein expression of α-7nAchR from lung tissue was assayed by western blot. **(A)** Representative images of HE-stained lung sections under light microscope in each group, the black arrow points to pulmonary vessels, and the enlarged vessel is presented in the upper left corner. **(B)** Statistical analysis of pulmonary arterial WT%. Pulmonary vessels with external diameters smaller than 200 μm were selected in each field (*n* = 6). **(C)** Statistical analysis of AchE activity in plasma (*n* = 6). **(D)** Statistical analysis of AchE activity in lung tissue (*n* = 6). **(E)** Relative protein expression of α-7nAchR in lung tissue (*n* = 3). AchE, acetylcholinesterase; α-7nAchR, nicotinic acetylcholine receptor alpha 7; Ctrl, control; DON, donepezil; HE, hematoxylin and eosin; MCT, monocrotaline; WT, wall thickness. *P < 0.05, vs. Ctrl group, ^#^*P* < 0.05, vs. MCT group.

To determine the effects of DON on pulmonary arterial remodeling, the thickness of the pulmonary artery was examined under a light microscope. Histological analyses of pulmonary arterioles showed an increased pulmonary arterial wall thickness (WT, %) from 22.3% in the control group to 63.0% in the MCT group, which decreased to 41.0% after DON treatment (*P* < 0.05, Figures 1A,B). This finding indicates that DON significantly reduced pulmonary arterial remodeling.

To evaluate changes of the cholinergic system, AchE activity in plasma and lung tissue was evaluated. DON significantly reduced AchE activity in the plasma and lung tissue of MCT rats by 2.1- and 2.5-fold, respectively (all *P* < 0.05, Figures 1C,D). This result indicates that DON effectively enhance the parasympathetic activity by inhibiting AchE activity in PAH.

To determine how parasympathetic activity exerts its protective effect against PAH, the relative expression of the cholinergic receptor α-7nAchR in lung tissue was detected. Consistently, the expression of α-7nAchR in the lung tissue of MCT rats was significantly decreased by 3.9-fold after DON treatment (*P* < 0.05, Figure 1E). These results suggest that DON enhances parasympathetic activity to exerts its beneficial function by suppressing α-7nAchR expression. Correspondingly, the heart rate of MCT-induced rats after DON treated was also significantly reduced, suggesting a DON-suppressed parasympathetic activity (Supplementary Figure 2).

### DON Improved Hemodynamics and RV Dysfunction in the Rats With MCT-Induced PAH

**Figure 2:**
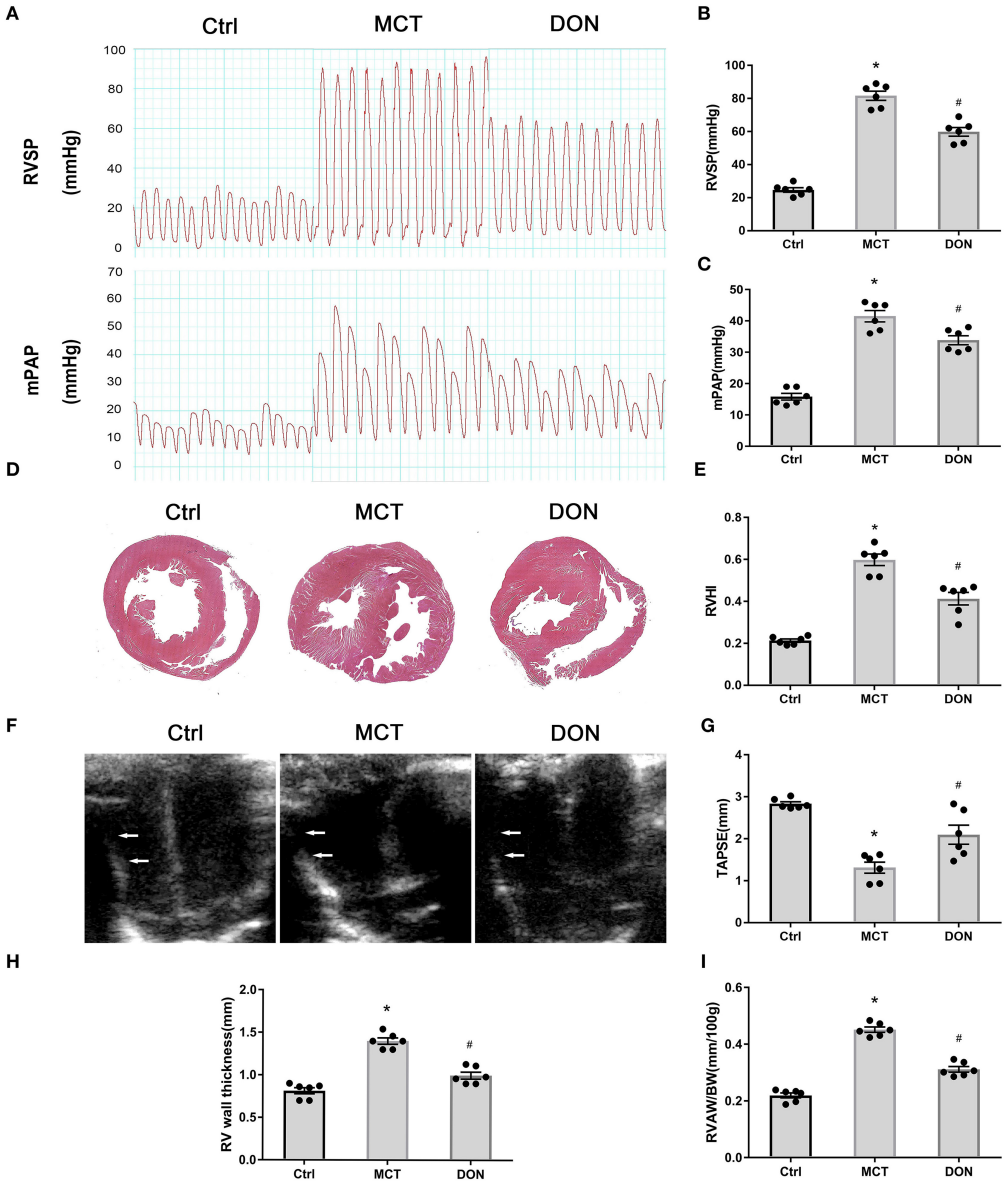
DON improves hemodynamics and RV dysfunction in MCT-induced PAH rats. At the end of the study, RVSP and mPAP were measured with closed-chest technique to show the hemodynamic changes of MCT-induced rats after DON treated. The heart of rats was fixed and stained with HE to show the RV expanding and remodeling. RVHI indicates the ratio of RV weight to LV + S weight. Transthoracic echocardiography at the apical 4-chamber view to show the indicators of RV remodeling and dysfunction. **(A)** RVSP and mPAP measurement in experimental rats. **(B)** Statistical analysis of RVSP. **(C)** Statistical analysis of mPAP. **(D)** Representative HE staining of heart samples. **(E)** Quantitative analysis of RVHI. **(F)** Transthoracic echocardiography at the apical 4-chamber view. **(G)** Statistical analysis of TAPSE. **(H)** Statistical analysis of RV wall thickness. **(I)** Statistical analysis of the ratio of RVAW to BW. BW, body weight; Ctrl, control; DON, donepezil, HE, hematoxylin and eosin; LV, left ventricle; MCT, monocrotaline; mPAP, mean pulmonary arterial pressure; RVSP, right ventricular systolic pressure; RV, right ventricle; RVHI, RV hypertrophy index; RVAW, right ventricular anterior wall; S, septum; TAPSE, tricuspid annular plane systolic excursion. *P < 0.05, vs. Ctrl group, ^#^*P* < 0.05, vs. MCT group (*n* = 6).

To assess hemodynamics changes, RV systolic pressure (RVSP) and mean pulmonary arterial pressure (mPAP) have been measured by RV catheterization at the end of the study (Figure 2A). RVSP and mPAP in the MCT group, respectively, reached 82 and 42 mmHg, which were significant compared to that in the control group (*P* < 0.05, Figures 2B,C). However, RVSP and mPAP significantly decreased to 60 and 34 mmHg, respectively, after DON treatment (*P* < 0.05, Figures 2B,C). Accordingly, the survival rate and body weight of rats were also increased in DON group when compared with MCT group (Supplementary Figure 3; Supplementary Table 1), which may result from the improved RV dysfunction.

Histologic analyses of heart specimens and RV sections were performed. RV wall dilation was found to have increased in the MCT-treated rats, while DON significantly reversed this effect (Figure 2D). RV hypertrophy index (RVHI) is calculated from the ratio of the weight of RV to the weights of LV plus S, which is an indicator of the degree of cardiac hypertrophy and RV remodeling. In our result, RVHI was markedly increased to 0.60 in MCT-induced PAH rats when compared with the control rats of 0.22; however, it was significantly declined to 0.41 after treated with DON (*P* < 0.05, Figure 2E). These results suggested that DON improved circulatory hemodynamics and RV remodeling in the rats with MCT-induced PAH.

Echocardiography was used to measure the RV wall thickness followed by TAPSE to further confirm RV function (Figure 2F). RV thickness increased from 0.8 mm in the control group to 1.4 mm in the MCT group, while it reduced to 1.0 mm after DON treatment. The indicator of RV thickness/body weight more accurately reflected RV hypertrophy and remodeling. TAPSE is a well-indicator of RV contractile function, which significantly decreased in the rats with MCT-induced PAH, while it reversed after DON treatment (all *P* < 0.05, Figures 2G–I). DON effectively reversed the indicators of RV remodeling and dysfunction, which was likely because of increased pulmonary arterial pressure.

### DON Reversed the Proliferation and Apoptotic Resistance of PASMCs in the Rats With MCT-Induced PAH

**Figure 3:**
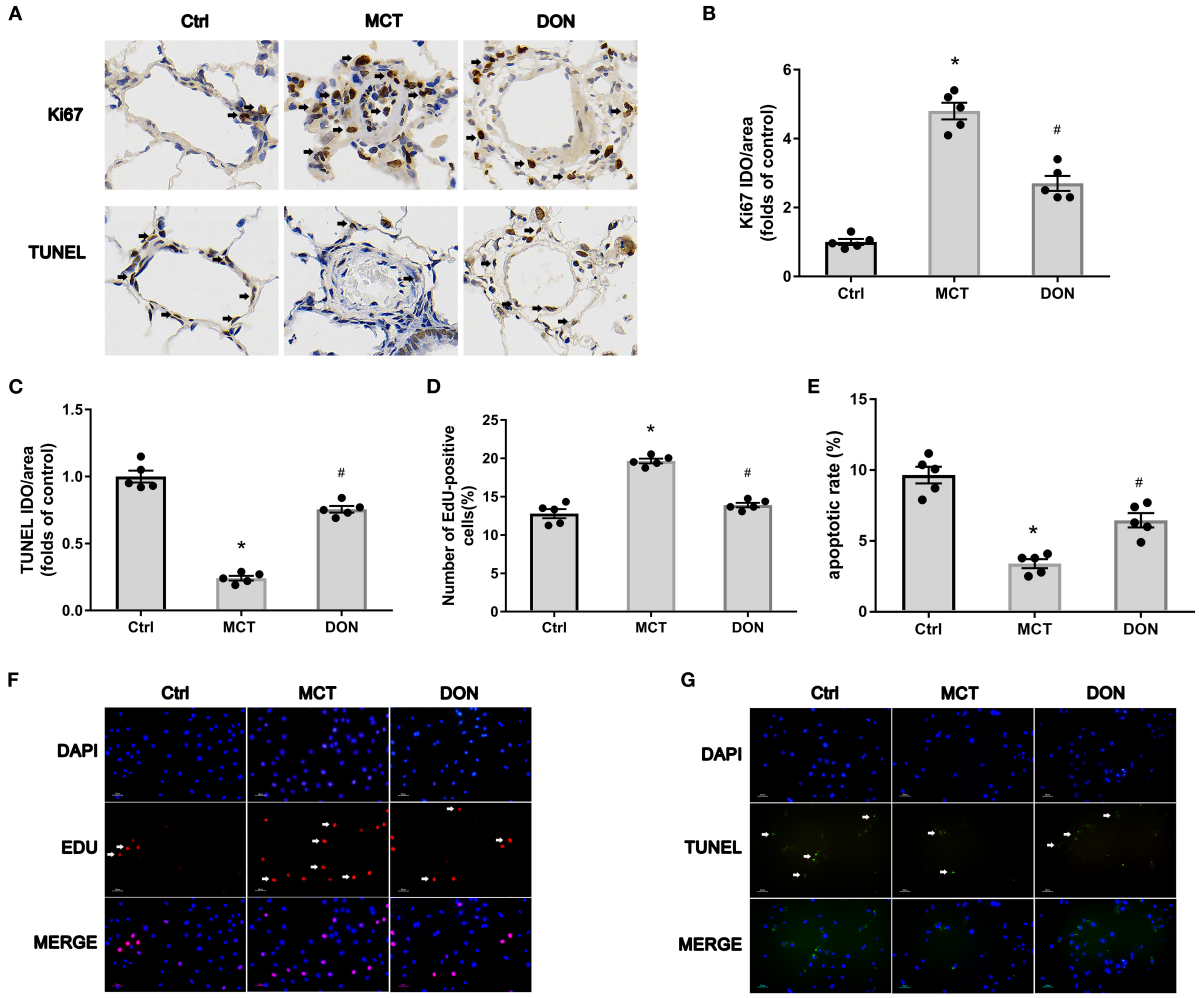
DON reverses PASMC proliferation and apoptosis resistance in MCT-induced PAH rats. Immunohistochemical staining for Ki67 and TUNEL in lung tissue to show the effect of DON on PASMC proliferation and apoptosis in MCT-induced rats *in vivo* (×400 magnification). Relative positive cells of the pulmonary artery were counted. To further test PASMC proliferative ability *in vitro*, PASMCs in the Ctrl, MCT, DON groups were isolated and cultured. The proliferative and apoptotic abilities of PASMCs were, respectively, assayed by immunofluorescence staining with EdU and TUNEL. The ratio of EdU and TUNEL positive cells to total cell numbers per high power field (×200 magnification) were calculated. **(A)** Representative immunohistochemical staining for Ki67 and TUNEL of pulmonary artery samples. **(B)** Quantitative analysis of Ki67 positive rate. **(C)** Quantitative analysis of TUNEL positive rate. **(D)** Statistical analysis of the EdU+ cell rate. **(E)** Statistical analysis of the apoptosis rate of PASMCs. **(F)** Representative immunofluorescent staining for EdU of PASMCs. **(G)** Representative immunofluorescent staining for TUNEL of PASMCs. Ctrl, control; DON, donepezil; DAPI, 4',6-diamidino-2-phenylindole; EdU, 5-ethynyl-20-deoxyuridine; MCT, monocrotaline; OD, optical density; PASMCs, pulmonary artery smooth muscle cells; TUNEL, terminal deoxynucleotidyl transferase dUTP nick end labeling. *P < 0.05, vs. Ctrl group, ^#^*P* < 0.05, vs. MCT group (*n* = 5).

To explore the mechanisms underlying the effects of DON in terms of reduction in the thickened pulmonary arterial wall in PAH, the proliferation and apoptosis of PASMCs were evaluated. Ki67 immunohistochemical staining of lung tissue sections was used to evaluate the effect of DON on the proliferative ability of PASMCs in MCT- induced PAH rat model. The percentage of Ki67-positive cells around the pulmonary artery was significantly increased to 4.8-fold in the MCT group; however, it was reduced to 2.7-fold after DON treatment (*P* < 0.05, Figures 3A,B), suggesting that DON markedly inhibited PASMC proliferation *in vivo*.

Besides, TUNEL immunohistochemical staining of lung tissue sections was used to evaluate the effect of DON on the apoptosis of PASMCs in MCT induced PAH rat model. The percentage of TUNEL-positive cells were decreased 4.1-fold in the MCT group when compared with control group, but increased 3.1-fold in the DON group (*P* < 0.05, Figures 3A,C). These results indicate that DON markedly promotes PASMC apoptosis in MCT-induced PAH model.

To further confirm this effect, primary PASMCs were isolated from the three groups and cultured *in vitro*. EdU staining was performed to evaluate the proliferative ability of PASMCs. The percentage of EdU+ cells increased from 12.8% in the control group to 19.7% in the MCT group (*P* < 0.05, Figures 3D,F). On the other hand, after DON treatment, EdU+ cells significantly decreased to 13.9% compared with that in the MCT group (*P* < 0.05, Figures 3D,F). This result showed the robust proliferative ability of PASMCs in PAH, which was obviously suppressed after DON treatment.

PASMCs were subjected to TUNEL assay to determine the effect of DON on their apoptotic ability. The apoptosis rate of PASMCs in the control group rats was 9.7%, which decreased to 3.4% in the MCT group (*P* < 0.05, Figures 3E,G). However, the apoptosis rate increased to approximately 6.5% in the DON group, which was highly significant compared to that in the MCT group (*P* < 0.05, Figures 3E,G). This result suggested that DON effectively promoted the apoptosis of PASMCs in PAH.

### DON Reduced Pulmonary Inflammation in the Rats With MCT-Induced PAH

**Figure 4:**
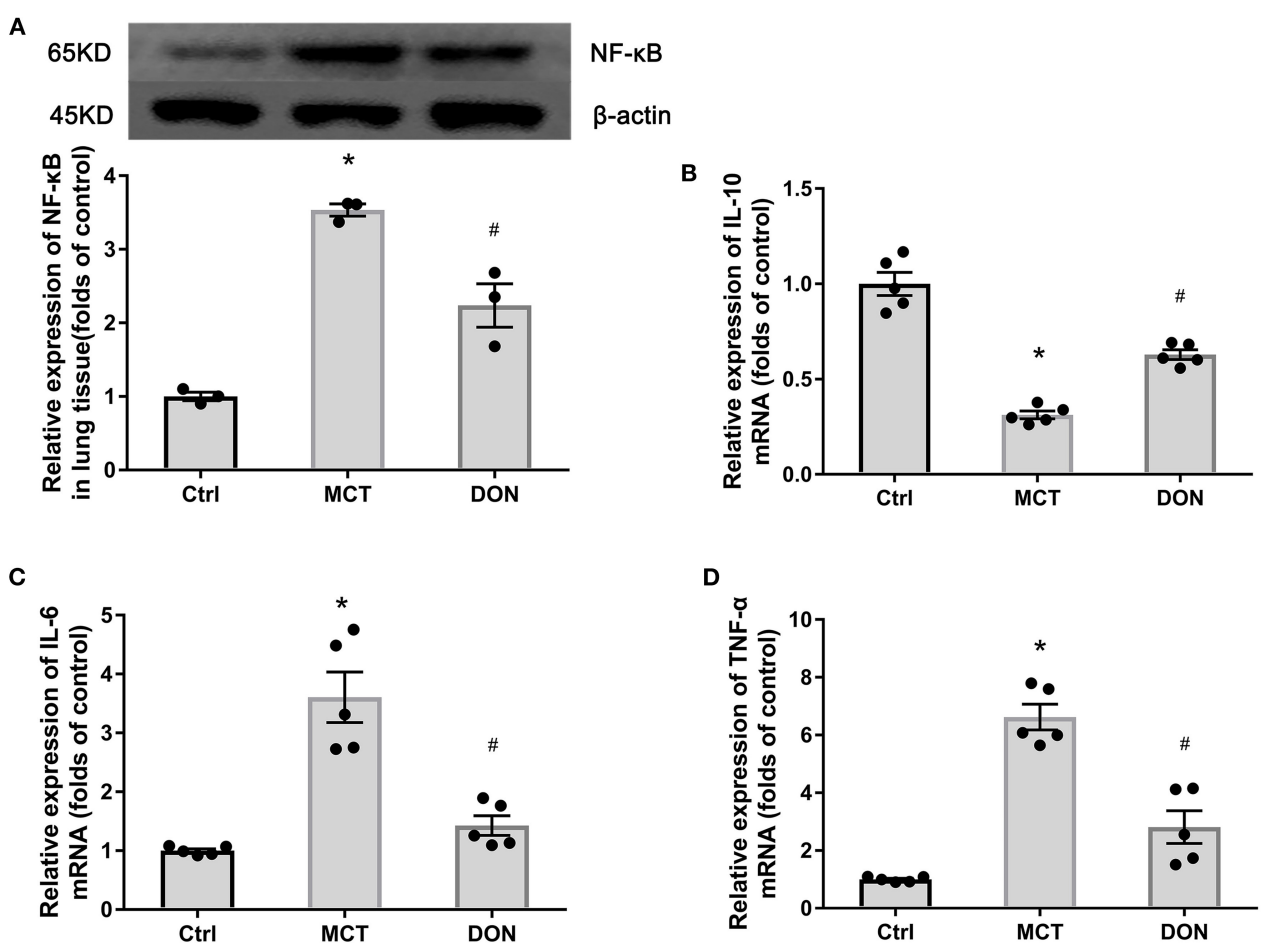
DON reduced pulmonary inflammation. Relative protein expression of NF-κB in lung tissue in MCT-induced rats treated with DON were tested by western blot. Relative expression of inflammatory mRNA in lung tissue were detected by real time-PCR. **(A)** Relative protein expression of NF-κB (*n* = 3). **(B)** Quantitative analysis of relative mRNA expression of IL-10 (*n* = 5). **(C)** Quantitative analysis of relative mRNA expression of IL-6 (*n* = 5). **(D)** Quantitative analysis of relative mRNA expression of TNF-α (*n* = 5). Ctrl, control; DON, donepezil; IL-6, interleukin-6; IL-10, interleukin-10; MCT, monocrotaline; NF-κB, nuclear factor-κB; TNF-α, tumor necrosis factor-α. *P < 0.05, vs. Ctrl group, ^#^*P* < 0.05, vs. MCT group.

To assess the inflammation of local pulmonary tissue in the rats with MCT-induced PAH, the expression of NF-κB in lung tissue was detected. The relative expression of NF-κB increased to 3.5-fold in the lung tissue of the rats in the MCT groups compared to that in the rats in the control group. On the other hand, NF-κB expression decreased by 1.6-fold after DON treatment (*P* < 0.05, Figure 4A). This result indicates enhanced local inflammation in the lung in PAH, which was suppressed after DON treatment.

To further elucidate the anti-inflammatory effects of DON, the expression of anti-inflammatory cytokine IL-10 and proinflammatory cytokines IL-6, TNF-α in pulmonary tissues were measured. The relative mRNA expression of IL-10 decreased 3.2-fold in lung tissue of the MCT group, while the relative mRNA expression of IL-6 and TNF-α increased to 3.6- and 6.6-fold, respectively, compared to that in the control group (all *P* < 0.05, Figures 4B–D). However, after DON administration, the relative mRNA expression of IL-10 increased by 2.0-fold, while that of IL-6 decreased by 2.6-fold, that of TNF-α decreased by 2.4-fold, compared to the corresponding levels in the MCT group (all *P* < 0.05, Figures 4B–D). This finding suggests that DON exerted anti-inflammatory effects in the rats with MCT-induced PAH.

### DON Suppressed Pulmonary Macrophage Infiltration as Well as M2-Macrophage Activation

**Figure 5:**
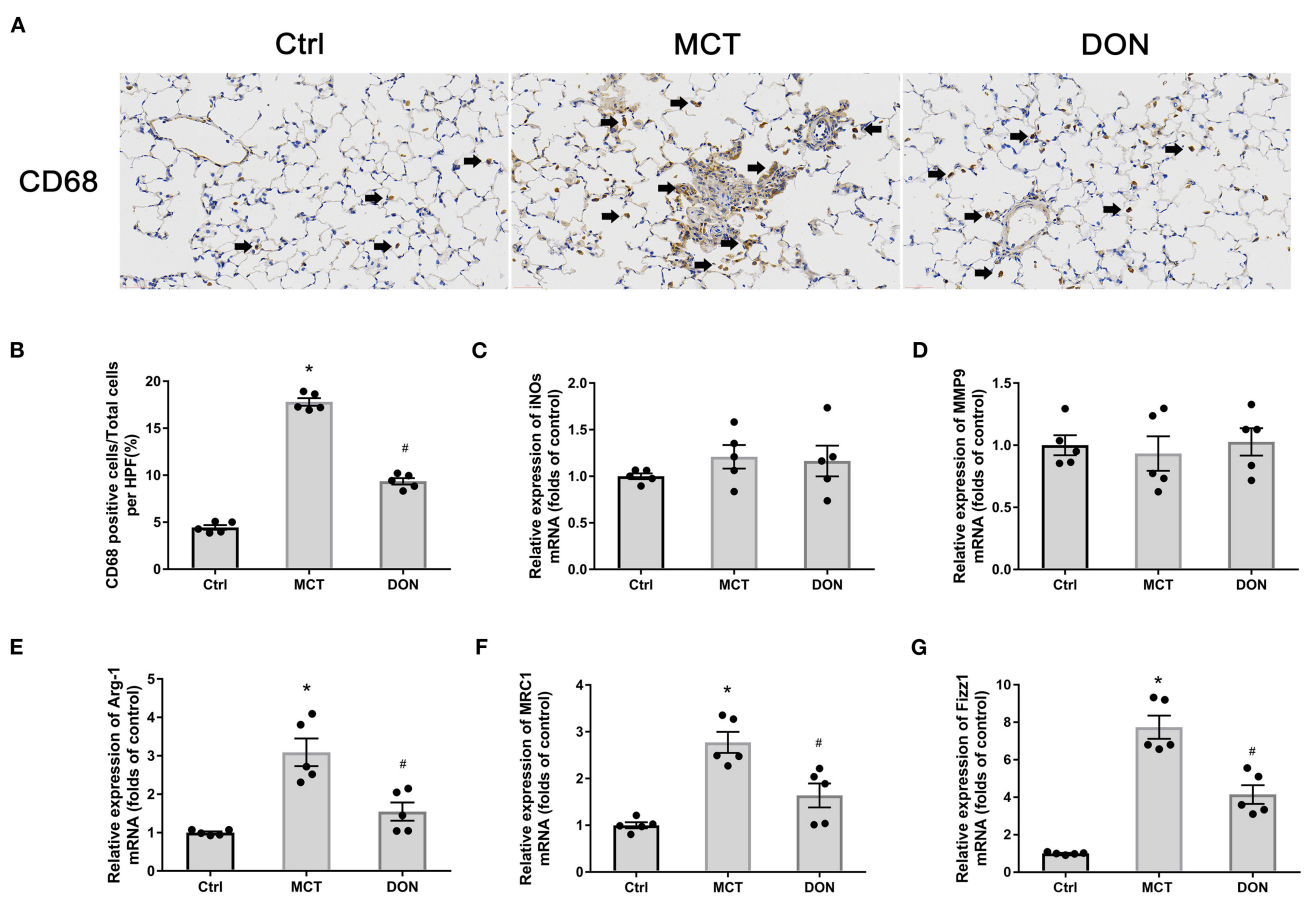
DON reduced macrophage activation in the lung. Immunohistochemical staining for CD68 antibody in lung tissue to show the effect of DON on macrophages infiltration in MCT-induced rats, which were scanned and analyzed by DSAssistantLite software (Motic, China; ×400 magnification, image size: 715 × 408 μm). The positive staining rate are counted with the mean of 5 random fields. The biomarkers of M1- and M2-macrophage in lung tissue were tested by real time-PCR. **(A)** Representative immunohistochemical staining for CD68. **(B)** Quantitative analysis of the CD68 positive cell rate. **(C)** Quantitative analysis of relative mRNA expression of iNOS. **(D)** Quantitative analysis of relative mRNA expression of MMP9. **(E)** Quantitative analysis of relative mRNA expression of Arg-1. **(F)** Quantitative analysis of relative mRNA expression of MRC1. **(G)** Quantitative analysis of relative mRNA expression of Fizz1. Arg-1, argirase-1; Ctrl, control; DON, donepezil; Fizz1, found in inflammatory zone 1; iNOs, inducible nitric oxide synthase; MCT, monocrotaline; MMP9, matrix metalloproteinase 9; MRC1, mannose receptor C1. *P < 0.05, vs. Ctrl group, ^#^*P* < 0.05, vs. MCT group (*n* = 5).

To evaluate the effects of DON on pulmonary macrophage infiltration in the rats with PAH, immunohistochemistry was performed for CD68 in the lungs (Figure 5A). The proportions of CD68 positive cells, representing macrophages in the lung tissue, increased 17.8% of the MCT-treated rats from 4.44% in the control group, while the proportions of these cells appeared to have decreased to 9.35% after DON treatment (all *P* < 0.05, Figure 5B). This result suggests that macrophage infiltration in lung tissue significantly increased in PAH, which could be reversed by DON treatment.

We also determined the phenotype of the macrophages that accumulated in pulmonary tissues. The characteristic biomarkers for M1-macrophages include iNOs and IL-6, MMP9, and those for M2-macrophages include Arg-1, MRC1, and Fizz1. Interestingly, the relative expression levels of iNOs and MMP9, in addition to IL-6, did not change significantly (Figures 5C,D). Conversely, the relative mRNA expression levels of Arg-1, MRC1, and Fizz1 increased 3.1, 2.8, and 7.7-fold, respectively, in the MCT group in comparison with the corresponding levels in the control group (all *P* < 0.05, Figures 5E–G). However, after DON treatment, the relative mRNA expression levels of Arg-1, MRC1, and Fizz1 reduced 2.1, 1.7, and 1.9-fold in comparison with those in the MCT group (all *P* < 0.05, Figures 5E–G). These results suggest that DON mainly suppressed inflammation and M2-macrophage activation in MCT-induced rats.

### DON Reduced the Proportion and Activation of M2-Macrophages in BMDMs

**Figure 6:**
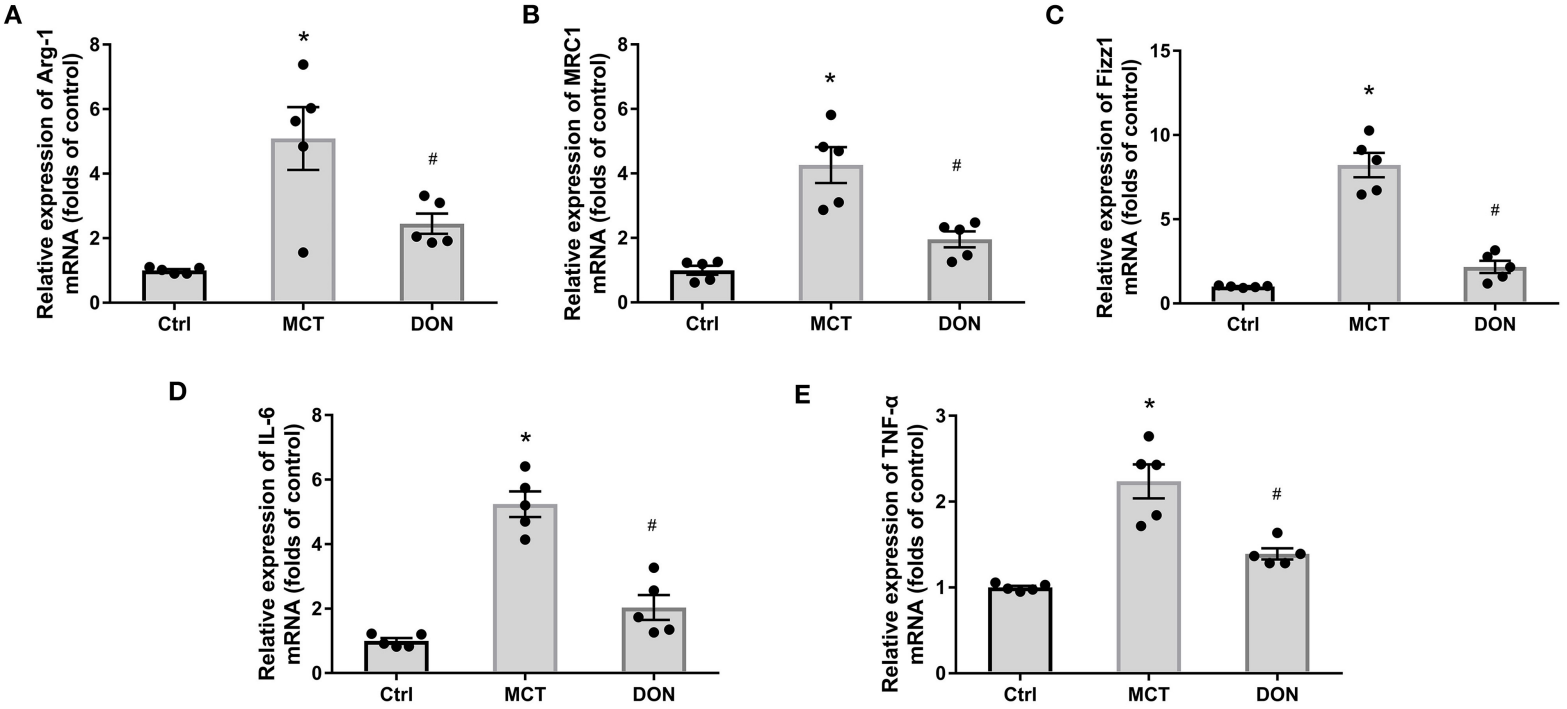
DON reduced M2-macrophage phenotype in BMDMs. BMDMs from the three groups were isolated. After purified and cultured, the biomarkers of M2-macrophage and inflammatory factors of BMDMs were identified by real time-PCR. **(A)** Quantitative analysis of relative mRNA expression of Arg-1. **(B)** Quantitative analysis of relative mRNA expression of MRC1. **(C)** Quantitative analysis of relative mRNA expression of Fizz1. **(D)** Quantitative analysis of relative mRNA expression of IL-6. **(E)** Quantitative analysis of relative mRNA expression of TNF-α. Arg-1, argirase-1; BMDMs, bone marrow-derived macrophages; Ctrl, control; DON, donepezil; Fizz1, found in inflammatory zone 1; IL-6, interleukin-6; MCT, monocrotaline; MRC1, mannose receptor-1; TNF-α, tumor necrosis factor-α. *P < 0.05, vs. Ctrl group, ^#^*P* < 0.05, vs. MCT group (*n* = 5).

To verify the effects on M2-macrophage activation in PAH rats, BMDMs were isolated and cultured, and the biomarkers of M2-macrophages were assayed. The relative mRNA expression levels of Arg-1, MRC1, and Fizz1, respectively, increased 5.0, 4.3, and 8.2-fold in the BMDMs of MCT-induced rats in comparison with the corresponding levels in control rats (all *P* < 0.05, Figures 6A–C). However, the relative mRNA expression levels of Arg-1, MRC1, and Fizz1 decreased 2.1, 2.2, and 3.9-fold, respectively, in the DON group in comparison with those in the MCT group (all *P* < 0.05, Figures 6A–C). These results further confirmed that DON represses M2-macrophage activation in rats with MCT-induced PAH.

To test the function of M2-macrophages after treatment with DON in MCT-induced rats, the relative mRNA expression levels of IL-6 and TNF-α were tested in BMDMs; the IL-6 level increased 5.2-fold and TNF-α levels increased 2.2-fold in comparison with the levels in the control group (all *P* < 0.05, Figures 6D,E). However, the relative mRNA expression levels of IL-6 and TNF-α decreased 2.6- and 1.6-fold, respectively, in the BMDMs after DON treatment in comparison with the levels in the MCT group (all *P* < 0.05, Figures 6D,E). This indicates that DON effectively suppressed the activation of M2-macrophages and further reduced its effect on promoting inflammatory factors release in MCT-induced PAH rats.

### BMDMs Treated With DON Inhibited PASMC Proliferation and Promoted Their Apoptosis

**Figure 7:**
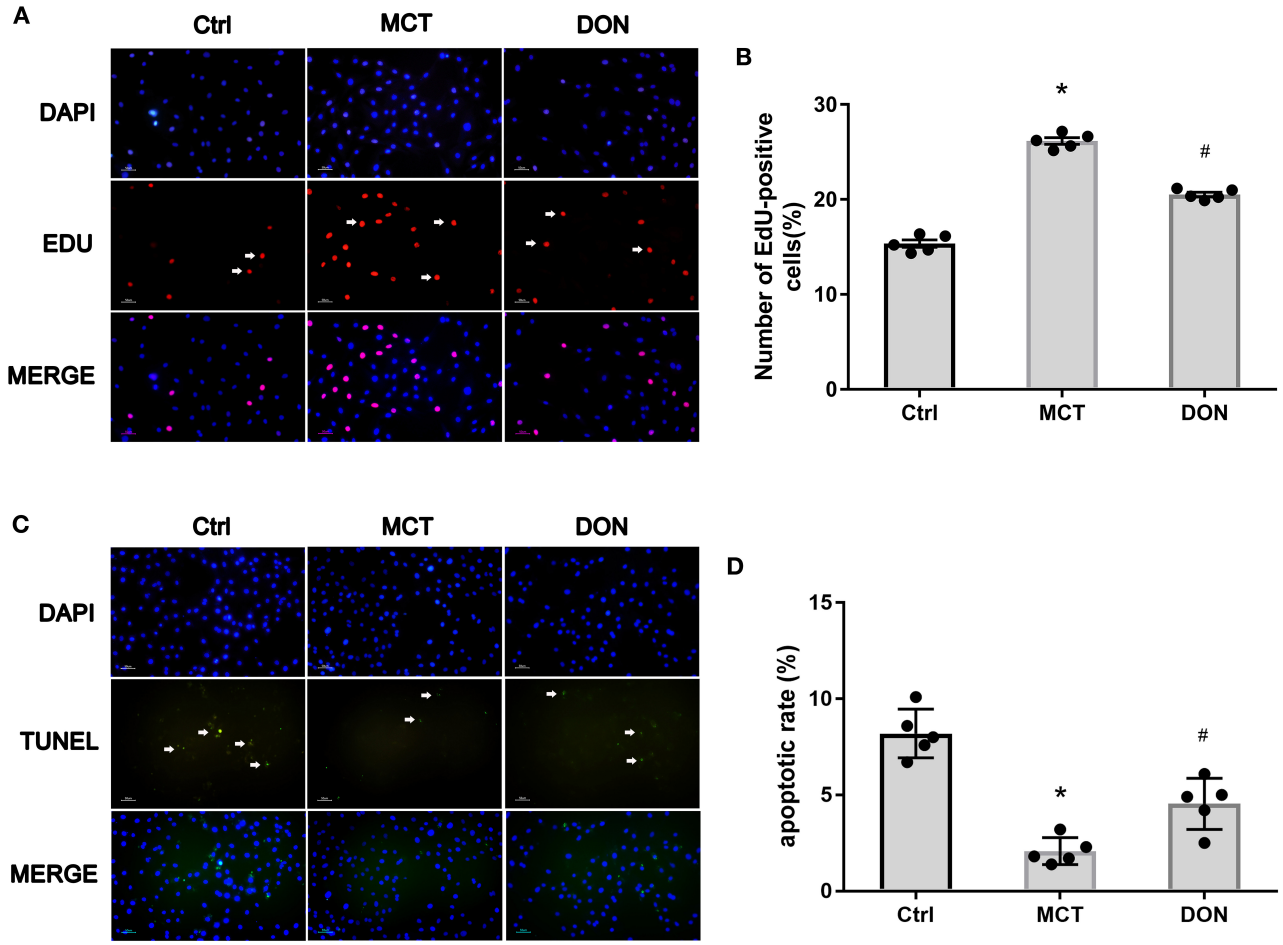
M2-macrophages inhibited PASMC proliferation and promoted its apoptosis. PASMCs in MCT-induced rats were isolated and cultured, which were co-cultured with the supernatant of BMDMs isolated from the Ctrl, MCT and DON group, respectively. After 24 h cultivation, the proliferative and apoptotic abilities of PASMCs were, respectively, assayed by EdU and TUNEL Assay Kit. The ratio of EdU and TUNEL positive cells to total cell numbers per high power field (×200 magnification) were calculated. **(A)** EdU stain to test proliferation of PASMCs. **(B)** Statistical analysis of EdU+ cell rate. **(C)** TUNEL assay to detect apoptosis of PASMCs. **(D)** Statistical analysis of apoptosis rate. BMDMs, bone marrow-derived macrophages; Ctrl, control; DON, donepezil; DAPI, 4',6-diamidino-2-phenylindole; EdU, 5-ethynyl-20-deoxyuridine; MCT, monocrotaline; PASMCs, pulmonary artery smooth muscle cells; TUNEL, Terminal deoxynucleotidyl transferase dUTP nick end labeling. *P < 0.05, vs. Ctrl group, ^#^*P* < 0.05, vs. MCT group (*n* = 5).

To evaluate the effects of M2-macrophages with or without DON treatment on PASMCs in rats with MCT-induced PAH, the EdU assay was used to assess the proliferative ability of PASMCs (Figure 7A). PASMCs from MCT rats were co-cultured with the supernatant of BMDMs in the control, MCT, and DON groups, respectively. After 24 h co-incubation, EdU staining was used to test PASMC proliferation. The percentage of EdU+ cells in the control group was 15.3%, and it increased to 26.2% in the MCT group and reduced to 20.5% after DON treatment (all *P* < 0.05, Figure 7B), indicating that DON exerts an effective anti-proliferative action on PASMCs in MCT rats by inhibiting M2-macrophage activation.

Consistent with these findings, the TUNEL assay was used to test the pro-apoptotic effects of DON on PASMCs by inhibiting M2-macrophage activation. After co-culture with the supernatant of BMDMs, the apoptosis rate of PASMCs was 8.2% in the control group, 2.1% in the MCT group, and 4.5% in the DON group (all *P* < 0.05, Figures 7C,D). These observations suggest that DON could reverse the abnormal functioning of PASMCs by regulating macrophage activation.

## Discussion

**Figure 8:**
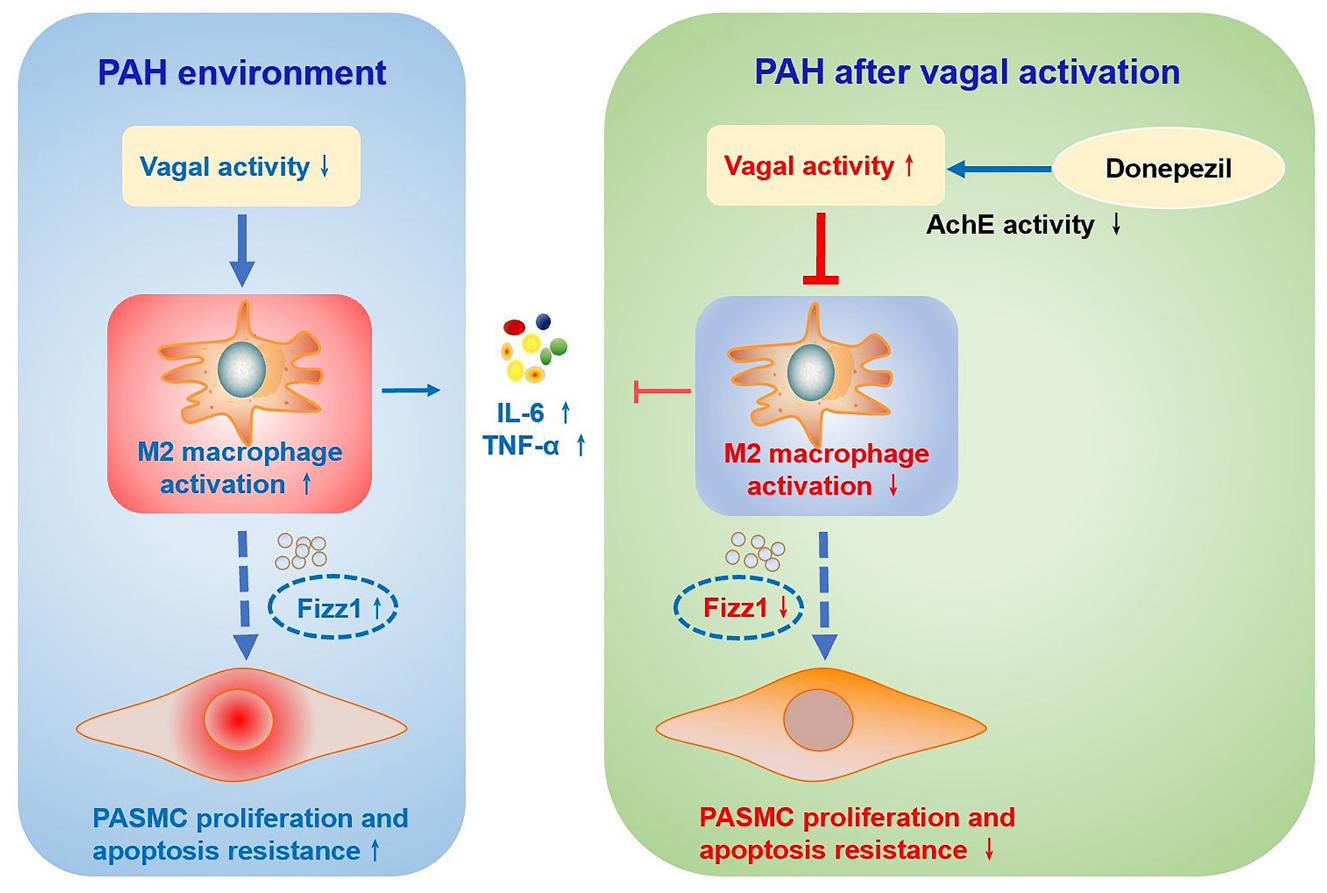
Schematic illustration of the effect of DON on ameliorating MCT-induced PAH by regulating M2 macrophage activation. In the MCT-induced PAH rat model, parasympathetic activity is reduced, inflammatory response and M2-macrophages are activated, and the expression of pro-inflammatory factors and pro-proliferation factor Fizz1 is increased, and subsequently increase PASMC proliferation and apoptosis resistance. However, DON enhances parasympathetic nerve activity by reducing AchE activity, and suppresses the inflammatory response and M2-macrophage activation, thereby inhibiting PASMC proliferation and promoting its apoptosis to reverse PAH, which is speculated to be related to decreased expression of Fizz1. AchE, acetylcholinesterase; Fizz1, found in inflammatory zone 1; IL-6, interleukin-6; MCT, monocrotaline; PAH, pulmonary arterial hypertension; PASMCs, pulmonary artery smooth muscle cells; TNF-α, tumor necrosis factor-α.

This study showed that parasympathetic activation with DON effectively improved pulmonary vascular remodeling and RV dysfunction in rats with MCT-induced PAH. This DON-induced increase in parasympathetic activation manifested as decreased AchE activity and then targeted α-7nAchR to exert its biological effects. Mechanically, DON was found to inhibit PASMC proliferation and promote its apoptosis. Moreover, DON could alleviate the inflammatory response and M2-macrophage activation in the lung tissue of PAH rats. Importantly, studies have illustrated that DON reversed PASMC dysfunction via suppressing M2-macrophage activation (Figure 8).

In this study, we found that parasympathetic activation via DON could effectively reverse the course of MCT-induced PAH. Previous studies have reported the beneficial effects of enhancing parasympathetic activity on PAH by non-invasive electrical stimulation or anticholinergic drugs (14–16, 33). These studies also outlined the effects of restoring autonomic balance by electrical vagal nerve stimulation in PAH rats, which could effectively improve the survival of rats with hypoxia/SU5416-induced PAH (15). Moreover, another study clarified that pyridostigmine enhanced parasympathetic activity and reduced sympathetic activity, improving RV dysfunction and pulmonary vascular remodeling in rats with hypoxia/SU5416-induced PAH (16). Different from previous studies, we have tested protective effects of another cholinesterase inhibitor DON on MCT-induced PAH rat model. Besides, the MCT-induced PAH rat model was administrated with a relative lower drug dosage of 50 mg/kg to improve survival rate (34), which also different from other studies with a higher dosage of 60 mg/kg. Overall, the protective effects of parasympathetic activation on PAH are consistent with the findings of previous studies. Although we considered that DON may be more effective to enhance the parasympathetic activity for it possessing the properties of an independent anti-inflammatory effects (35, 36).

Acetylcholine is the primary neurotransmitter for parasympathetic nerves to exert their protective effect, and it is degraded by AchE, which binds to its nicotinic and muscarinic receptors (37). According to our result, DON significantly repressed the AchE activity in both plasma and lung tissue of MCT rats, suggesting that intraperitoneal injection 1 mg/kg DON effectively corrected the downregulation of parasympathetic activity in PAH, whose dosage function has been evidenced in previous studies (22, 23, 38, 39). We also showed that DON treatment obviously reduced α-7nAchR expression in the lung tissue of PAH rats. One of the present explanations for these findings is that they are consistent with an adaptive, albeit inadequate, response to reduced parasympathetic signaling (16). Moreover, previous studies have proved that α-7nAchR was the main factor mediating the anti-inflammatory and anti-fibrotic effects of parasympathetic activation (16, 40). Thus, α-7nAchR is considered to be the main receptor for DON to exert its beneficial effects in rats with MCT-induced PAH.

We found that DON significantly reversed pulmonary artery remodeling and PASMC dysfunction. Fundamentally, the excessive proliferation and apoptotic resistance of PASMCs is essential for vascular remodeling in PAH (41). Chen et al. (42) demonstrated the effects of the vagus nerves on the proliferation and apoptosis of lung stem cells via regulation of α-7nAchR-mediated fibroblast growth factor 10 (FGF10) production. This finding suggested the presence of a parasympathetic nerve protective mechanism for lung injury due to regulation of the proliferation and apoptosis of cells (42). Furthermore, a recent study has discussed the anti-proliferative effects of parasympathetic activation on PAH, but it did not report the proliferative function of PASMCs (16). In contrast to the previous results, our findings described the direct anti-proliferation and pro-apoptotic effects after parasympathetic activation on PAH by assessing the proliferative and apoptotic ability in PASMCs.

PAH is characterized by robust proinflammatory cytokine infiltration and a perivascular inflammatory response (43, 44). We found that DON markedly reduced the inflammatory response and proinflammatory cytokines in the lung tissue of PAH rats, including reduced protein expression of NF-κB and secretion of the inflammatory factors IL-6 and TNF-α, as well as increased secretion of the anti-inflammatory factor IL-10. Generally, parasympathetic activation exerts its function mainly via anti-inflammation and anti-fibrotic mechanisms. Previous studies reported that enhancement of parasympathetic activity significantly alleviated pulmonary perivascular inflammation and reversed pulmonary artery remodeling by down-regulating the levels of the proinflammatory cytokines IL-6, IL-1b, TNF-α, and MCP-1 in the lung tissue of rats with MCT-induced PAH (14, 15). Thus, DON exerts its beneficial function in PAH via an anti-inflammation route.

We further found that the level of the macrophage biomarker CD68 was reduced in the lung tissue of MCT-induced rats after DON treatment, indicating reduced pulmonary macrophage infiltration in DON group rats. This is consistent with the findings of previous studies, in which extensive infiltration of macrophages around the pulmonary perivascular region was observed in various animal models (45, 46). More importantly, the findings suggested that M2-macrophage activation plays a more predominant role in PAH development than M1-macrophage activation via regulation of profibrotic and proinflammatory effects (11). In this study, we also found that DON could inhibit M2-macrophage activation in PAH by detecting its biomarkers and secreting factors, such as Arg-1, MRC1 and Fizz1. Previous studies have reported the cardioprotective effect of parasympathetic activation in cardiovascular disease, which are related to the regulation of M2-macrophage function (18, 19). However, this is the first study to report the actions of parasympathetic activation to inhibit M2-macrophage activation in rats with MCT-induced PAH.

We also found that M2-macrophages could inhibit the proliferation of PASMCs and promote their apoptosis. Vergadi et al. (47) were the first to emphasize the important role of M2-macrophage-induced pulmonary vascular remodeling in the progression of hypoxia-induced PAH. Abid et al. (20) further observed the function of PASMCs in idiopathic PAH patients after co-culture with murine macrophages, in which the M2-macrophage phenotype was polarized by IL-4 *in vitro*. Their results showed that M2-macrophages reversed the proliferation and migration of PASMCs in hypoxia/SU5416-induced PAH by inhibiting CCR2 and CCR5 collaboration. More rigorously, we directly found that DON could mediate PASMC function by inhibiting M2-macrophage activation, suggesting a novel mechanism and a potential therapeutic approach for PAH.

To further reveal the mechanisms underlying M2-macrophage-regulated PASMC dysfunction, we observed that Fizz1 showed the most significant changes in our result. More importantly, Fizz1, also called hypoxia-induced mitotic factor, is believed to have potent mitogenic, angiogenic, and vasoconstrictive effects, which has also been studied in PAH (48). A previous study discovered that Fizz1 induced PASMC proliferation and migration by down-regulating the expression of the calcium-binding protein S100A11 in PAH patients (49). Moreover, by knocking out or overexpressing the Fizz1 gene in the rodent PAH model, Lin et al. (50) recently validated that Fizz1 mediated the crosstalk between PAECs and PASMCs, which promoted the alteration of PASMCs to a proliferative phenotype and pulmonary vascular remodeling in PAH. Considering the function and characteristics of Fizz1 in promoting PASMC proliferation in PAH, further studies will explore whether Fizz1 secretion is responsible for mediating the crosstalk between macrophage and PASMCs.

## Conclusion

PAH is a life-threatening disease with a poor prognosis and has no effective treatment. Correction of autonomic nerve imbalance has been suggested to significantly suppress inflammation, improve pulmonary arterial and RV remodeling, and subsequently ameliorate PAH progression. Our findings demonstrated that the AchE inhibitor DON protects PAH by resisting inflammation and pulmonary vascular remodeling. More importantly, the suppression of M2-macrophage activation and consequent reversal of PASMC proliferation and apoptotic resistance has been first identified as the main beneficial mechanism of DON in PAH. Our findings provide more protective evidence for enhancing parasympathetic activity via reduced pulmonary inflammation and pulmonary arterial remodeling to offer a more potent therapeutic strategy for PAH.

## Limitation

Our study had some limitations. First, there are limitations to identify α-7nAchR as the main receptor for cholinergic anti-inflammation, for we did not block α-7nAchR to test the biological effects of DON. Besides, although referenced previous studies in isolating and culturing the BMDMs, we have failed to test its biomarkers via flow cytometry. In addition, our finding only showed that Fizz1 was the most significantly changed M2-macrophage-derived factor. However, we did not silence or overexpress the Fizz1 gene and then verify its effects on PASMCs. Therefore, further studies are needed to validate whether Fizz1 secretion is responsible for DON against PASMC dysfunction by changing its expression. Moreover, other potential mechanisms of M2-macrophages in mediating the function of PASMCs also need to be investigated.

## Data Availability Statement

The original contributions presented in the study are included in the article/Supplementary Material, further inquiries can be directed to the corresponding author/s.

## Ethics Statement

The animal study was reviewed and approved by the Animal Research Committee of Central South University in Hunan, China. Written informed consent was obtained from the owners for the participation of their animals in this study.

## Author Contributions

YG designed the study, acquired funding, and administrated the project. HQ and YZ performed the experiments. PJ analyzed the data. HQ drafted the manuscript. ZL, SG, YH, and YG critically revised the manuscript and supervised the study. All authors contributed to the article and approved the final version of the manuscript for publication.

## Conflict of Interest

The authors declare that the research was conducted in the absence of any commercial or financial relationships that could be construed as a potential conflict of interest.
