# Supplementary material for: Donepezil Ameliorates Pulmonary Arterial Hypertension by Inhibiting M2-Macrophage Activation
Source: Front Cardiovasc Med. 2021 Mar 15;8:639541. doi: 10.3389/fcvm.2021.639541 (PMC8005547; doi:10.3389/fcvm.2021.639541)
Supplement: Supplementary file 1 [file Data_Sheet_1.pdf]

Supplementary Material:

Donepezil Ameliorates Pulmonary Arterial Hypertension by Inhibiting M2-Macrophage Activation

Haihua Qiu[1], Yibo Zhang[2], Zhongyu Li[3], Ping Jiang[1], Shuhong Guo[1], Yi He[1], Yuan Guo[1, 4]*

[1] Department of Cardiovascular Medicine, The Affiliated Zhuzhou Hospital Xiangya Medical College, Central South University, Zhuzhou, Hunan 412000, China.

[2] Department of Ultrasound, The Affiliated Zhuzhou Hospital Xiangya Medical College, Central South University, Zhuzhou, Hunan 412000, China.

[3] Laboratory Medicine Center, The Affiliated Zhuzhou Hospital Xiangya Medical College, Central South University, Zhuzhou, Hunan 412000, China.

[4] Department of Cardiovascular Medicine, Xiangya Hospital, Central South University, Changsha, Hunan 410008, China.

Corresponding Author:

Yuan Guo, E-mail: guoyuan8141@csu.edu.cn.

Short title: Donepezil and Pulmonary Arterial Hypertension

Keywords: Cholinesterase inhibitor, donepezil, M2-macrophage, pulmonary arterial hypertension, pulmonary arterial smooth muscle cells.

1. Materials and methods

1.1. Identification of PASMCs

After purified to passage 2 and confluence to 80-90%, pulmonary arterial smooth muscle cells (PASMCs) were undergoing immunofluorescence staining with α-SMA. Briefly, the cells were fixed with 4% paraformaldehyde and permeabilized in 0.2% Triton X-100. After blocking with 1% BSA, the cells were incubated with primary antibody of α-SMA, (Abcam, Cambridge, MA, USA) at 4°C overnight. The cells were then incubated with Alexa Fluor 555-conjugated donkey anti-rabbit secondary IgG (A0453, Beyotime, China). Fluorescence was observed with an immunofluorescence microscope (×400 magnification, Olympus Corp, Tokyo, Japan).

1.2. Indexes of routine observation of experimental animals

The male Sprague Dawley rats were randomly divided into three groups and the body weight was recorded at the beginning of experiment that induced by a single intraperitoneal injection (50 mg/kg) of monocrotaline (MCT). After then body weight has been tested weekly until the end of donepezil (DON) treatment. Before weighing the rats, they have been fasted for 8 hours. The dead rats were recorded during the experiment and the survival rate has been calculated at the end of study. Heart rate of rats was also recorded and finally analyzed by an electrocardiograph (ECG) instrument (Chengdu instrument factory, China).

2. Figures

Figure S1. Identification of PASMCs with immunohistochemical staining of α-SMA.

Representative immunohistochemical staining of PASMCs with α-SMA under fluorescent inverted microscope (magnification×400, Olympus Corp, Tokyo, Japan). After purified to passage 2 and confluence to 80-90%, PASMCs were underwent immunofluorescence staining with α-SMA and the positive cells could be defined as PASMCs.

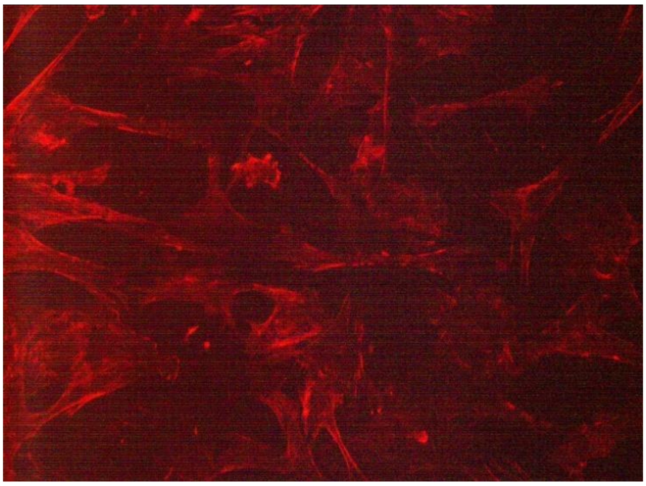

Figure S2. DON reduced heart rate of MCT-induced PAH rats.

Statistical analysis of heart rate. At the end of the study, heart rate was recorded and analyzed by an ECG instrument. Heart rate in the MCT treated mice was significantly increased, which was decreased by DON intervention. Ctrl: control, DON: donepezil, MCT: monocrotaline. *P < 0.05, versus Ctrl group, $^{\#}P$ < 0.05, versus MCT group (n=5).

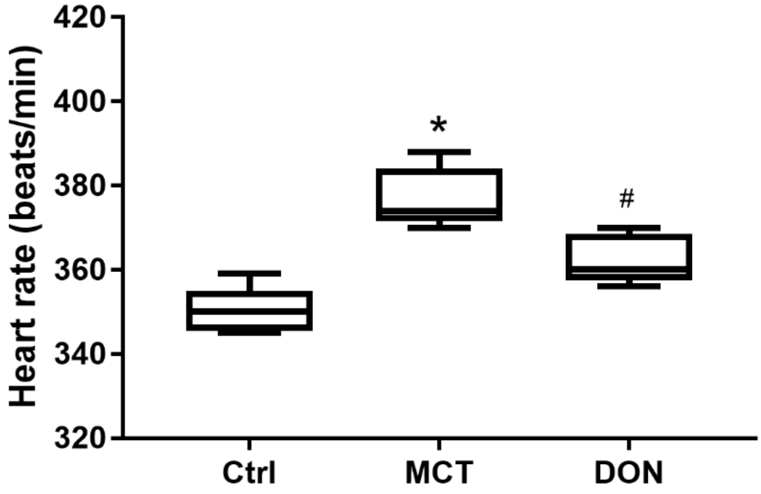

Figure S3. DON improved survival rate of MCT-induced PAH rats.

Survival curve of MCT-induced PAH rats that treated with DON. Survival rate has been analyzed at the end of the study using Prism for Windows (GraphPad 8 Software). The survival rate of DON group was significantly higher than the MCT group (P < 0.05). Ctrl: control, n=15, DON: donepezil, n=15, MCT: monocrotaline, n=15.

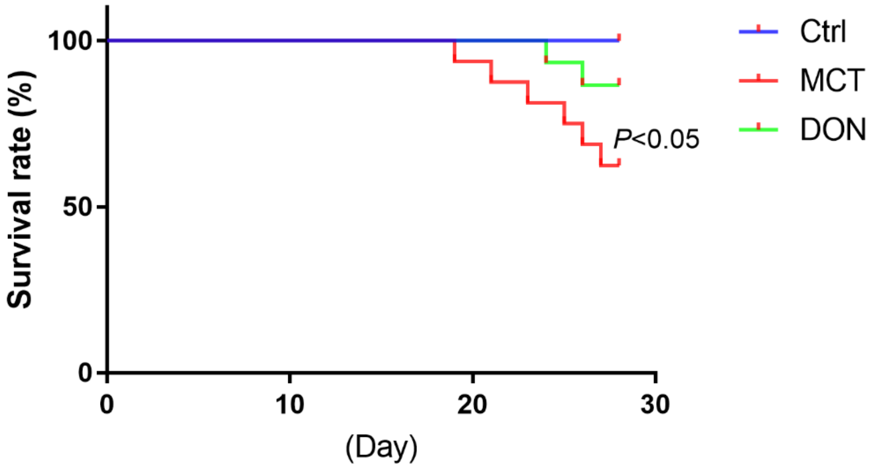

Table S1. DON increased body weight of MCT-induced PAH rats.

	Ctrl (g)	MCT (g)	DON (g)
0 day	209.0±6.65	204.6±4.62	210.6±6.93
7 day	258.6±11.38	234.1±6.28	239.9±13.14
14 day	301.3±16.82	261.9±12.56	273.5±20.42
21 day	340.5±24.17	291.6±19.65*	309.5±17.56
28 day	360.1±25.68	281.7±40.12*	312.8±28.09$^{\#}$

Body weight of rats was recorded weekly during the experiment and it has been expressed as mean ± SD. Differences were tested by using one-way analysis of variance, followed by Tukey-Kramer test. Ctrl: control, n=15, DON: donepezil, n=15, MCT: monocrotaline, n=15. *$P < 0.05$, versus Ctrl group, $^{\#}P < 0.05$, versus MCT group.
